# A unified Internet-delivered exposure treatment for undifferentiated somatic symptom disorder: single-group prospective feasibility trial

**DOI:** 10.1186/s40814-022-01105-0

**Published:** 2022-07-19

**Authors:** Jonna Hybelius, Anton Gustavsson, Sandra af Winklerfelt Hammarberg, Eva Toth-Pal, Robert Johansson, Brjánn Ljótsson, Erland Axelsson

**Affiliations:** 1 Department of Psychology, Stockholm University, Stockholm, Sweden; 2 Liljeholmen Primary Health Care Center, Region Stockholm, Liljeholmstorget 7, Stockholm, 117 63 Sweden; 3 Academic Primary Health Care Center, Region Stockholm, Stockholm, Sweden; 4 Division of Family Medicine and Primary Care, Department of Neurobiology, Care Sciences and Society, Karolinska Institutet, Huddinge, Sweden; 5 Division of Psychology, Department of Clinical Neuroscience, Karolinska Institutet, Stockholm, Sweden

**Keywords:** Behavioural medicine, Behaviour therapy, Feasibility studies, Internet-based intervention

## Abstract

**Background:**

Exposure-based psychological treatment appears to have beneficial effects for several patient groups that commonly report distress related to persistent somatic symptoms. Yet exposure-based treatment is rarely offered in routine care. This may be because existing treatment protocols have been developed for specific symptom clusters or specific unwanted responses to somatic symptoms, and many clinics do not have the resources to offer all these specialised treatments in parallel. In preparation for a randomised controlled trial, we investigated the feasibility of a new and unified Internet-delivered exposure treatment (OSF.io: cnbwj) for somatic symptom disorder regardless of somatic symptom domain (e.g. cardiopulmonary, fatigue, gastrointestinal, pain), combination of unwanted emotions (e.g. anger, anxiety, fear, shame) and whether somatic symptoms are medically explained or not. We hypothesised that a wide spectrum of subgroups would show interest, that the treatment would be rated as credible, that adherence would be adequate, that the measurement strategy would be acceptable and that there would be no serious adverse events.

**Methods:**

Single-group prospective cohort study where 33 self-referred adults with undifferentiated DSM-5 somatic symptom disorder took part in 8 weeks of unified Internet-delivered exposure treatment delivered via a web platform hosted by a medical university. Self-report questionnaires were administered online before treatment, each week during treatment, post treatment and 3 months after treatment.

**Results:**

Participants reported a broad spectrum of symptoms. The Credibility/Expectancy mean score was 34.5 (SD = 7.0, range: 18–47). Participants completed 91% (150/165) of all modules and 97% of the participants (32/33) completed at least two exposure exercises. The average participant rated the adequacy of the rationale as 8.4 (SD = 1.5) on a scale from 0 to 10. The post-treatment assessment was completed by 97% (32/33), and 84% (27/32) rated the measurement strategy as acceptable. The Client Satisfaction Questionnaire mean score was 25.3 (SD = 4.7, range: 17–32) and no serious adverse events were reported. Reductions in subjective somatic symptom burden (the Patient Health Questionnaire 15; *d* = 0.90) and symptom preoccupation (the somatic symptom disorder 12; *d* = 1.17) were large and sustained.

**Conclusions:**

Delivering a unified Internet-delivered exposure-based treatment protocol for individuals with undifferentiated somatic symptom disorder appears to be feasible.

**Trial registration:**

ClinicalTrials.gov, NCT04511286. Registered on August 13, 2020.

**Supplementary Information:**

The online version contains supplementary material available at 10.1186/s40814-022-01105-0.

## Key messages regarding feasibility

- Prior to this feasibility trial it was unclear whether a unified Internet-delivered exposure-based treatment could be offered for undifferentiated DSM-5 somatic symptom disorder, without stratification on any particular somatic symptom domain or type of unwanted emotional response, with the unified rationale for exposure being found credible, overall satisfaction being adequate and patients reporting clinically relevant improvement and acceptable adverse events.
- We found that individuals with a wide spectrum of persistent physical symptoms showed interest, rated the treatment as credible and adhered to the study protocol. The participants reported large reductions in subjective somatic symptom burden and symptom preoccupation, without serious adverse events.
- Delivering a unified Internet-delivered exposure-based treatment for undifferentiated somatic symptom disorder appears to be feasible. This motivates further evaluation in the relevant routine care contexts such as primary care, and also further evaluation of how much various patient groups have to gain from being enrolled in an exposure-based treatment developed for their specific somatic disease or syndrome (such as anxiety in asthma, atopic dermatitis, atrial fibrillation and so on) rather than a unified treatment. In the planned randomised controlled trial, we may consider another way of identifying individuals with distress related to persistent somatic symptoms that does not depend on the patient’s reaction being deemed “disproportionate” or “excessive” as this often proved difficult to determine. There could also be benefits of a longer treatment.

## Background

Heightened reactivity and the preoccupation with somatic symptoms appear to produce an increase in subjective somatic symptom burden and long-term disability in many health conditions [1–3]. When such a preoccupation with symptoms is deemed to be excessive, patients are likely to meet full criteria for Diagnostic and Statistical Manual of Mental Disorders 5 (DSM-5) somatic symptom disorder (SSD) [4]. Although little is understood of the mechanisms by which psychological factors continuously influence the experience, development and impact of somatic symptoms, many empirically informed models show sizeable overlap [5–12]. A common presumption is that unwanted responses—commonly emotions—can become associated with physical symptoms or related phenomena, and that such unwanted responses commonly imply changes in physiology and information processing that contribute to an increased subjective somatic symptom burden over time [13–16]. There is also good reason to believe that avoidance behaviours—that is, behaviours intended to minimise exposure to somatic symptoms or related phenomena in the short term (e.g. the avoidance of movement likely to bring about pain)—often contribute to maintaining or even exacerbating unwanted responses over time by preventing the acquisition of beneficial information (e.g. that movement does not result in catastrophic consequences), by hampering beneficial physiological processes (e.g. when more passivity leads to poor fitness and muscle weakness) and by resulting in a more restricted behavioural repertoire where the frequency and intensity of behaviours contingent on symptoms lead to heightened disability and reduced quality of life [17–19].

A common method of reducing avoidance behaviour and addressing unwanted learned responses is exposure therapy. In this type of treatment, the patient willingly engages with stimuli that give rise to unwanted physical sensations or unwanted responses in order to achieve therapeutic effects. Conventional exposure treatment as conceived in the cognitive-behavioural therapies is a structured intervention where the patient systematically identifies potential triggers for symptoms and discomfort and actively engages in exercises to evoke these triggers while refraining from strategies to reduce discomfort in the short term, and does this repeatedly in real life situations in the attempt to achieve therapeutic long-term effects [20]. Therapies with an emphasis on this type of conventional exposure exercises have been found to be efficacious in several conditions where patients commonly report distress related to somatic symptoms. In anxiety in asthma, within-group effects have been large on asthma control (*d* = 1.27), large on catastrophising, fear and avoidance (*d* = 0.90–1.52), and small to moderate on quality of life (*d* = 0.40) [11]. In atopic dermatitis, superiority has been seen on subjective somatic symptoms versus rudimentary education (between-group *d* = 0.75) and within-group effects have been large on subjective somatic symptoms (*d* = 0.93–1.09) and moderate to large on quality of life (*d* = 0.65–0.83) [21]. In paroxysmal atrial fibrillation, superiority on atrial fibrillation quality of life has been seen versus rudimentary education (between-group *d* = 0.77) and within-group effects have been small to large on subjective symptom severity (*d* = 0.46–0.91), large on cardiac anxiety (*d* = 1.43–1.83) and large on atrial fibrillation quality of life (*d* = 0.80–1.55) [22, 23]. In pain conditions including chronic back pain and fibromyalgia, superiority has been seen on composite syndrome severity versus a waiting-list (*d* = 0.90) [24], controlled effects versus other cognitive-behavioural therapies have been mixed [25, 26] and within-group effects have varied from small to large on pain (*d* = 0.35–1.01), moderate to large on pain-related reactivity including anxiety and catastrophising (*d* = 0.69–1.53) and moderate to large on disability (*d* = 0.46–1.77) [24–28]. In severe health anxiety, superiority on health anxiety has been seen versus a waiting-list (*d* = 0.80–1.27) [29], attention control (*d* = 1.62) [30] and behavioural stress management (*d* = 0.26) [31] but not cognitive restructuring techniques [32, 33] and within-group effects of interventions involving a therapist have been moderate to large on subjective somatic symptoms (*d* = 0.54–1.07), large on health anxiety and anxiety sensitivity (*d* = 0.95–1.76) and moderate to large on disability (*d* = 0.59–0.94) [29, 32–34]. In hyperacusis, superiority on audiological sensitivity has been seen versus a waiting-list (*d* = 0.67–0.69) and within-group effects have been moderate on audiological sensitivity (*d* = 0.51–0.56) and moderate on disability (*d* = 0.46) [35]. In irritable bowel syndrome, superiority in composite syndrome severity has been seen versus waiting-lists [36, 37] and stress management [38, 39] and within-group effects have been large on composite severity (*d* = 0.98–1.80) moderate to large on gastrointestinal-specific anxiety (*d* = 0.59–1.26) and moderate to large on quality of life (*d* = 0.40–1.02) [36–40]. In summary, exposure-based therapies have been found to have beneficial effects in many health conditions where distress related to somatic symptoms is common. However, to our knowledge, a unified exposure-based treatment that can be easily tailored to suit a wide spectrum of patients who experience distress related to somatic symptoms has never been evaluated empirically. Considering that many clinics, notably primary care clinics, are expected to serve a broad spectrum of patients, a unified treatment would often be easier to disseminate. Given the comorbidity between conditions where patients report distress related to somatic symptoms, many would probably also benefit from addressing several somatic symptom domains in the same exposure-based treatment [41, 42].

In this study, we aimed to assess the feasibility of delivering a unified exposure-based treatment intended to suit a wide spectrum of individuals with distress related to somatic symptoms warranting a diagnosis of DSM-5 somatic symptom disorder, regardless of precise somatic symptom domain (e.g. cardiopulmonary, fatigue, gastrointestinal, pain), combination of unwanted emotions (e.g. anger, anxiety, fear, shame) and whether somatic symptoms are medically explained or not. The treatment was delivered as a guided Internet-based therapy which is a proven format [43] that reduces the time needed from the therapist, makes interaction more flexible in time and place, and lowers the threshold for treatment-seeking in conditions where stigma is common. In accordance with study design guidelines [44, 45], we wanted to determine (1) if a reasonably wide spectrum of individuals with different forms of distress related to different somatic symptoms would show interest, (2) if at least 60% of treatment modules would be completed and at least 50% of participants would complete at least 2 exposure exercises, (3) if participants would rate the treatment as credible and find the rationale for exposure acceptable and relevant, (4) if at least 75% of patients would rate the measurement strategy as less than 7 on a scale from 0 (“Not at all stressful/bothering”) to 10 (“Extremely stressful/bothering”) and if at least 70% would complete the post-treatment assessment, (5) if the mean 8-item Client satisfaction questionnaire would be at least 22, (6) if at least moderate within-group effects, around *d* = 0.50, would be seen on subjective somatic symptom burden and symptom preoccupation and (7) if the frequency and severity of adverse events and negative experiences would be acceptable in light of the apparent efficacy.

## Methods

### Design

This was a single-group prospective cohort study designed to assess the feasibility of a unified Internet-delivered exposure therapy for undifferentiated DSM-5 somatic symptom disorder. Study site was Karolinska Institutet, a medical university in Stockholm, Sweden, and the protocol was approved by the Swedish ethical review authority (2020-01740). The study was preregistered on August 13, 2020 (ClinicalTrials.gov NCT04511286). We originally aimed to recruit 40 participants for 80% power in two-sided tests of moderate effects (*d* = 0.5) on efficacy outcomes measured at two time points, given a 5% alpha and 15% missing data at post-treatment. Within the feasibility framework, our line of reasoning was that should this study not be indicative of at least moderate within-group effects, it would not be feasible to study the treatment protocol further, and proceed to test for causal effects in a randomised controlled trial. In light of the high data retention (see below), prior to the data analysis, we ended the recruitment with 33 participants included in November 2020. All outcomes are reported in accordance with the 2010 CONSORT extension for feasibility trials [45].

### Participants

Participants were self-referred via the study website. We advertised the study via social media and at Liljeholmen Primary Health Care Center in Stockholm, under the heading “Are you bothered much by your physical symptoms? Have your symptoms taken control of your life?”. The ad made clear that individuals could be included in the study if they suffered from distress related to at least one somatic symptom such as “pain, gastrointestinal problems, palpitations, respiratory changes, urinary problems, menstrual cramps, impaired sexual functioning, dizziness, nausea, vision anomalies, smell anomalies, taste anomalies, hearing anomalies, a ‘lump in the throat’, sweating, eczema, itching, tremor, poor balance, or fatigue”. All applicants provided informed consent and completed a screening battery via the study web platform (two-factor authentication, encrypted traffic). A psychiatric telephone interview was then held with a last year master-level psychologist student (JH, AG) or PhD level clinical psychologist (EA) to assess the study eligibility criteria based on the Health preoccupation diagnostic interview (HPDI) [46] and the Mini international neuropsychiatric interview version 7 (MINI) [47]. The students were introduced to the assessment of SSD and then received continuous supervision, by the PhD level clinical psychologist (EA) who specialises in the somatic symptom and related disorders and similar phenomena. Though we did not evaluate inter-rater reliability in a formal manner, such an evaluation has been undertaken as part of a previous project [46] where, albeit in a slightly different setting, as in the present project the HPDI was used in conjunction with the MINI and this was found to result in acceptable inter-rater reliability in the assessment of SSD. In the present study, assessments were continuously reviewed by the PhD level clinician, and whenever further medical evaluation was deemed necessary, an experienced general practitioner (SWH) was consulted. We included adult Swedish citizens (≥ 18 years) with a principal diagnosis of DSM-5 SSD who expressed interest in psychological treatment, were able to read and write Swedish and completed the pre-treatment assessment. We did not require the SSD diagnosis to be based on any particular type of physical symptoms or any particular psychological response over and above the formal SSD criteria listed in the DSM-5. Thus, patients could be bothered by any combination of somatic symptoms, these symptoms could be medically explained or not, and if the patient reported excessive time and energy devoted to symptoms or health outcomes (criterion B3), this could take many forms. Worthy of notice, the SSD B criteria have attracted criticism because they require clinicians to assess whether the patient’s thoughts, feelings and behaviours are “excessive” or “disproportionate”. In this study, we interpreted the patient’s behaviour as excessive or disproportionate to the extent that we deemed it likely to lead to more harm than good in terms of suffering and functional impairment in the moderate to long term. Applicants were excluded if their preoccupation with somatic symptoms was better explained by another psychiatric condition such as illness anxiety disorder, panic disorder or body dysmorphic disorder. Applicants were also excluded if they suffered from a severe psychiatric condition such as bipolar disorder or a psychotic disorder, reported suicidal ideation, reported alcohol or substance use that would be a clear obstacle to therapy or were deemed not able to participate fully in exposure therapy without significant medical risks. Participants were required not to have made changes to continuous psychotropic medication in the past 4 weeks, to be willing to maintain the same dose during treatment and not to plan being absent for more than 1 week of the treatment period.

### Unified Internet-delivered exposure therapy

The Internet-delivered exposure therapy lasted 8 weeks and was a unified treatment similar to those evaluated for specific somatic conditions and specific types of symptom preoccupation (see the Background). This was a therapist-guided online self-help treatment, with most content conveyed by means of a text divided into five modules, reminiscent of book chapters. Each module came with homework exercises and questions for reflection. Communication with a therapist (JH, AG or EA) relied on an email-like system where the participants could expect a reply within two business days. Throughout the treatment, in order to promote adherence, therapists were encouraged to telephone participants who had been inactive for more than a few days on the online platform. Though therapists were not strictly prohibited from discussing the principles of treatment, most phone calls were brief and typically focused on practical problem solving such as logging in or making a schedule for working with the intervention. The idea was to steer the patient back to the treatment platform where a clear majority of the treatment content was conveyed. All therapists had formal training in behavioural interventions including exposure, and the students received regular supervision. The treatment was tailored to suit the needs of each participant, as based on functional analysis. Throughout the treatment, participants could follow three fictitious exemplar patients with various forms of distress related to symptoms: one with persistent pain and fatigue, one with cardiopulmonary symptoms and one with gastrointestinal symptoms. The treatment emphasised that symptom behaviours, i.e. behaviours that occur in response to distress and persistent somatic symptoms, and which serve to reduce distress or discomfort in the short to moderate term, are likely to have negative long-term effects. One reason given for this was that there are fewer opportunities for becoming better at managing difficult stimuli. Other reasons given were that discomfort and hypervigilance easily becomes generalised and that symptom behaviours can paradoxically worsen or bring about new symptoms. Last, symptom behaviours which increase the proportion of time devoted to somatic symptoms and health often make it more difficult to lead a fruitful life. The overarching rationale for exposure was that heightened reactivity to symptoms including changes in attention and physiological arousal is likely to lead to a higher subjective somatic symptom burden, and that exposure exercises are a method of changing such responses and increase the probability of having experiences that increase quality of life in the long term. Participants were encouraged to plan exposure exercises on a daily basis, while refraining from symptom behaviours. Module 1 introduced the treatment model and participants were encouraged to begin monitoring their symptom behaviours. In module 2, participants were encouraged to complete a series of interoceptive exposure exercises and to make a plan for response prevention. In module 3, participants continued with exposure and response prevention, with particular emphasis on exposure in vivo, i.e. “real life” situations tailored for the individual participant. In module 4, participants were encouraged to process their most unwanted thoughts related to symptoms by writing about unwanted outcomes coming true. Finally, in module 5, participants were encouraged to continue working with exposure and response prevention in a systematic manner. The treatment protocol, written in Swedish by EA, is free to use and available online (OSF.io identifier: cnbwj).

### Outcomes

Self-report questionnaires were administered online (i) at screening before the eligibility assessment, (ii) pre-treatment, (iii) every week during treatment, (iv) post-treatment and (v) 3 months after treatment. Subjective somatic symptom burden was assessed using the Patient Health Questionnaire 15 (PHQ-15) [48], in its original form at screening, and modified to concern the past week for all other measurement points. The PHQ-15 was also scored as four subfactors—cardiopulmonary, fatigue, gastrointestinal and pain [49]—each with a range of 0–2. We assessed symptom preoccupation using the Somatic Symptom Disorder 12 (SSD-12) [50], anxiety sensitivity using the 16-item version of the Anxiety Sensitivity Index (ASI-16) [51], health anxiety using the 14-item version of the Health Anxiety Inventory (HAI-14) [52], general anxiety using the GAD-7 [53], depression symptoms using the Patient Health Questionnaire 9 (PHQ-9) [54]. We assessed functional impairment using the 12-item World Health Organization Disability Assessment Schedule 2 (WD2-12) [55] with a range of 0–100 for the sum and subscales [56]. At week three, we assessed treatment credibility using a 5-item version of the Credibility/Expectancy scale [57] and the relationship with the therapist using a 6-item version of the Working Alliance Inventory (WAI-6) [31]. We assessed treatment satisfaction using the 8-item Client Satisfaction Questionnaire (CSQ-8) [58] and adverse events using free-text items where the respondent was instructed to describe up to three adverse events and rate how much this affected them at the time it occurred and at post treatment [34, 40]. We also administered the 20-item Negative Effects Questionnaire (NEQ-20) [59] which covers negative experiences in terms of increased symptoms, perceived insufficient quality of the treatment, dependency, stigma and hopelessness [60]. Last, we administered three items concerning covid-19 pandemic-related mood disturbance. See Supplement 1 for more information about these items and the subscales of the PHQ-15, WD2-12 and NEQ-20.

### Statistical analysis

For all feasibility outcomes except preliminary efficacy, a priori guidelines for interpretation were intended as crude rules of thumb and we did not employ inferential statistics. For the efficacy outcomes, we analysed change using linear mixed effects regression models fitted by maximum likelihood estimation using data from all 33 participants, with a spline at post-treatment so that distinct rates of change be modelled for the treatment and follow-up periods. We calculated standardised Cohen’s *d* effect sizes as the model-implied mean improvement divided by the pooled observed standard deviation of change over the corresponding time period. For Cohen’s *d*, absolute values of 0.8 are usually regarded as large, 0.5 as moderate and 0.2 as small [61]. We also determined the response rate on the PHQ-15 operationalised as a reduction of at least 2.3 points [62], using the fitted regression lines.

## Results

**Fig. 1:**
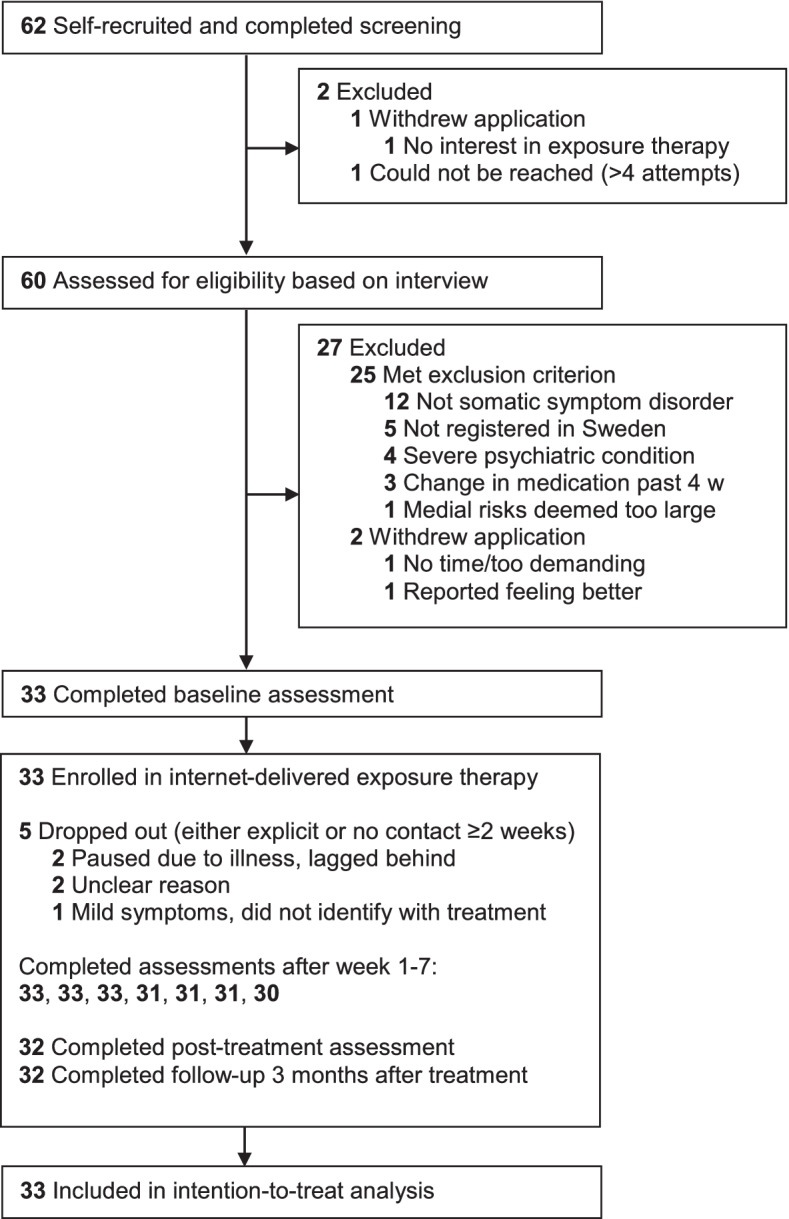
Flowchart of recruitment and participation

A study flowchart is presented in Fig. 1. The first participant was enrolled on September 11, 2020, and the last follow-up was completed on February 24, 2021. Sample characteristics are provided in Table 1. A typical participant was around 46 years old, female and highly educated and had suffered from SSD for 11 years with a moderate subjective somatic symptom burden [48] and high symptom preoccupation indicative of high health care use [50, 63].

**Table 1:** Participant characteristics before treatment

**Sociodemographic variables**	
Age in years	46 (14), 23–74
Female gender	22 (67%)
University education^a^	27 (82%)
Married or de facto	27 (82%)
Has children	26 (79%)
Employment	
Working full-time	21 (64%)
Working part-time (< 90%)	7 (21%)
Retired	4 (12%)
Student	1 (3%)
**Clinical variables**	
Somatic symptom disorder	
Somatic symptom burden (PHQ-15)^b^	11.8 (4.6), 3–20
Symptom preoccupation (SSD-12)^b^	33.2 (8.1), 22–47
Functional impairment (WD2-12)^b^	20.3 (16.1), 2.1–68.8
Age of onset	35 (18), 7–71
Psychiatric comorbidity, current	
Major depressive disorder	8 (24%)
Anxiety disorder, PTSD or OCD	15 (45%)
Non-psychiatric comorbidity, any time	
Hypertension	8 (24%)
Migraine	8 (24%)
Irritable bowel syndrome	7 (21%)
Osteoarthritis	6 (18%)
Atrial fibrillation	5 (15%)
Hiatal hernia	5 (15%)
Hyper- or hypothyroidism	5 (15%)
Atopic dermatitis	4 (12%)
Cancer, any	4 (12%)
Asthma	3 (9%)
Fibromyalgia	1 (3%)
Psoriasis	1 (3%)
Renal disease	1 (3%)
Medication	
Psychotropic	15 (45%)
Pain, prescribed	3 (9%)
**Recruitment path**	
Searching on the internet	15 (45%)
Social media platform	6 (18%)
Friend, acquaintance or family member	3 (9%)
Routine care clinician	2 (6%)
Other or does not remember	7 (21%)

Estimates are *n* (%) or M (SD), range. Psychiatric comorbidity is based on a diagnostic telephone interview and non-psychiatric diagnoses given by a physician are self-reported. *OCD* Obsessive–compulsive disorder, *PHQ-15* Patient Health Questionnaire 15, *PTSD* Post-traumatic stress disorder, *SSD-12* Somatic Symptom Disorder 12, *WD2-12* 12-item World Health Organization Disability Assessment Schedule 2

^a^International Standard Classification of Education 1997 (ISCED-97) level 4 or higher

^b^This refers to the screening values, with conventional questionnaire phrasings (as opposed to revised phrasings to concern the past week only)

### Other treatments and interaction with the therapist

During treatment, two participants (6%) lowered their dose of at least one psychotropic medication. Three participants (9%) consulted a psychologist or psychiatrist, two (6%) met with a general practitioner and two (6%) met with a physiotherapist outside of the study. In this study, the mean number of therapist minutes per patient and week was 16.4 (SD = 13.6; median = 12.4, IQR = 19.4–7.6). On average, participants sent 10.5 (SD = 7.0) email-like messages and received 16.8 (SD = 4.8) from their therapist. One third of the sample (11/33) received at least one phone call from their therapist. Three participants (9%) received more than one call (2, 2, and 3 calls). At week 3, the mean WAI-6 score was 35.2 (SD = 5.7).

### Feasibility aspect 1: sample distribution of somatic symptoms

**Fig. 2:**
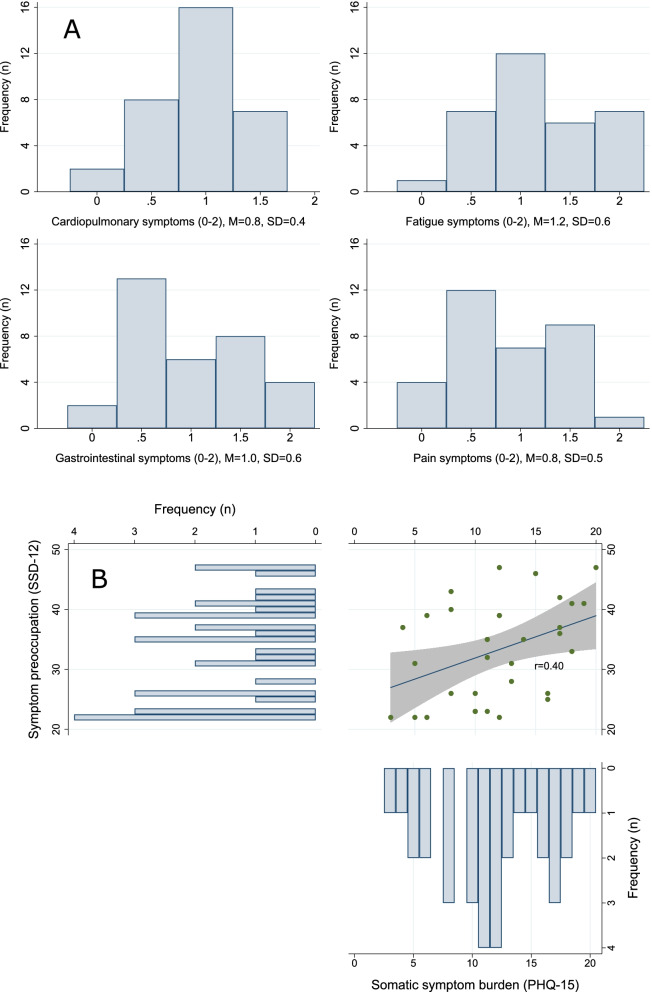
Subjective somatic symptom burden (**A**) and its relationship to symptom preoccupation (**B**) before treatment

Figure 2 illustrates the distribution of cardiopulmonary, fatigue, gastrointestinal and pain symptoms. Though on average each symptom domain was present to a moderate degree, there was also considerable variance. Correlations between the symptom domains were small to moderate (*r* = 0.08–0.38) and symptom preoccupation was moderately positively correlated with subjective somatic symptom burden (*r* = 0.40).

### Feasibility aspect 2: adherence to the treatment protocol

There were 150/165 (91%) completed modules; 60% being the prespecified threshold for adequacy. Participants reported completing an average of 28.4 (SD = 20.8) exposure exercises and 32/33 (97%) completed at least 2 exercises. The prespecified threshold was ≥ 50% of participants completing at least 2 exposure exercises, though this threshold was not intended as an ideal target, but rather a means of ensuring that the unified rationale approach to exposure would have to demonstrate some minimal promise for it to be pursued further in future studies.

### Feasibility aspect 3: treatment credibility and adequacy of the rationale

At treatment week 3, the mean score on the Credibility/Expectancy scale was 34.5 (SD = 7.0). At post-treatment, participants were asked about the rationale for exposure, i.e. how well the treatment’s unified description of behaviour, thoughts and emotions in relation to physical symptoms was adequate and applicable to their situation on a scale from 0 (“not at all relevant”) to 10 (“extremely relevant”) which resulted in mean score of 8.4 (SD = 1.5).

### Feasibility aspect 4: adequacy of measurement strategy

We collected 287/297 (97%) measurements over the treatment period. The post-treatment and 3-month assessments were each completed by 32/33 (97%) participants, the prespecified threshold for adequacy being 70%. Twenty-seven out of 32 (84%) found the assessment strategy to be acceptable (< 7, where 0 was “not at all stressful/distressing” and 10 “extremely stressful/distressing”; M = 3.0, SD = 2.9; median = 2, IQR = 5.5–0). The prespecified tolerability threshold was 75%.

### Feasibility aspect 5: satisfaction with treatment

The CSQ-8 mean score was 25.3 (SD = 4.7, *n* = 32), i.e. above the predefined guideline for adequacy which was a mean of 22. Four participants (13%) scored below 20 points. These participants reported stable or slightly worsened symptoms, two out of four reported having completed less than five exposure exercises, two out of four wrote that the treatment had been too text heavy and three out of four wrote that they had preferred to receive more support from the therapist (one participant mentioning face-to-face). In the sample as a whole, most participants indicated that the treatment met most or all of their needs (23/32, 47%; 8/32, 25%). One participant (3%) indicated that the treatment did not meet any needs. Most were willing to recommend the treatment to a friend (30/32, 94%).

### Feasibility aspect 6: efficacy outcomes

**Fig. 3:**
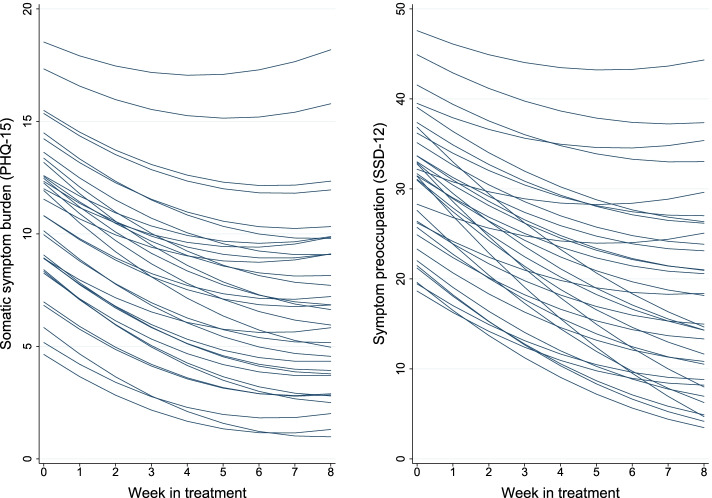
Spaghetti plots of fitted regression lines illustrating change in subjective somatic symptom burden (the Patient Health Questionnaire 15) and symptom preoccupation (the Somatic Symptom Disorder 12) during exposure-based treatment for undifferentiated somatic symptom disorder

The efficacy outcomes are tabulated in Table 2. There were large reductions in overall subjective somatic symptom burden (the PHQ-15; *d* = 0.90) and symptom preoccupation (the SSD-12; *d* = 1.17), and a curvilinear pattern in change, smaller towards the end of treatment (see Fig. 3). Though this study was conducted in the autumn of 2020, and there was an increase in covid-19 pandemic-related mood disturbance from pre- to post-treatment, this did not show a clear relationship with the reduction seen in subjective somatic symptom burden (see Supplement 1). The reduction in symptom preoccupation correlated strongly (*r* = 0.68) with the reduction in subjective somatic symptom burden. The intention-to-treat response rate [62] was 91% (30/33).

**Table 2:** Change in efficacy outcomes including subjective somatic symptom burden and symptom preoccupation

Outcome	Measure (theoretical range)	Pre-treatment			Post-treatment			3 months			Change Pre-Post		Change Pre-3MFU	
		M	SD	*n*	M	SD	*n*	M	SD	*n*	Slope (95% CI)	Cohen’s *d*^c^	Slope (95% CI)	Cohen’s *d*^c^
Subjective somatic symptom burden	PHQ-15 (0–30)^ab^	11.1	4.7	33	7.0	5.4	32	7.0	4.6	32	− 4.2 (− 5.5 to − 2.9)	0.90	− 4.2 (− 5.6 to − 2.8)	1.00
Cardiopulmonary symptoms	PHQ-15 subscale (0–2)^a^	0.7	0.5	33	0.4	0.4	32	0.4	0.3	32	− 0.3 (− 0.4 to − 0.2)	0.62	− 0.3 (− 0.4 to − 0.2)	0.65
Fatigue symptoms	PHQ-15 subscale (0–2)^a^	1.2	0.5	33	0.7	0.6	32	0.8	0.5	32	− 0.5 (− 0.7 to − 0.3)	0.75	− 0.3 (− 0.5 to − 0.2)	0.63
Gastrointestinal symptoms	PHQ-15 subscale (0–2)^a^	0.9	0.6	33	0.7	0.5	32	0.6	0.6	32	− 0.3 (− 0.5 to − 0.2)	0.56	− 0.3 (− 0.5 to − 0.2)	0.59
Pain symptoms	PHQ-15 subscale (0–2)^ab^	0.8	0.5	33	0.6	0.6	32	0.5	0.6	32	− 0.3 (− 0.4 to − 0.1)	0.44	− 0.3 (− 0.4 to − 0.1)	0.53
Symptom preoccupation	SSD-12 (0–48)^ab^	30.7	9.2	33	18.4	12.5	32	18.5	10.5	32	− 13.0 (− 16.5 to − 9.4)	1.17	− 12.8 (− 16.5 to − 9.1)	1.32
Anxiety sensitivity	ASI-16 (0–64)	26.4	10.8	33	17.1	10.0	32	17.4	11.1	32	− 9.5 (− 13.1 to − 6.0)	0.89	− 9.2 (− 12.9 to − 5.6)	0.84
Health anxiety	HAI-14 (0–42)	25.5	6.8	33	18.8	8.6	32	18.4	9.2	32	− 6.7 (− 9.3 to − 4.2)	0.94	− 7.2 (− 9.7 to − 4.7)	0.95
General anxiety	GAD-7 (0–21)	9.5	5.5	33	6.3	5.2	32	5.9	5.5	32	− 3.4 (− 5.0 to − 1.7)	0.73	− 3.7 (− 5.4 to − 2.1)	0.72
Depression symptoms	PHQ-9 (0–27)	7.9	5.6	33	4.9	4.8	32	5.1	5.1	32	− 3.2 (− 4.6 to − 1.8)	0.70	− 2.9 (− 4.4 to − 1.5)	0.77
Overall functional impairment	WD2-12 (0–100)	19.5	14.6	33	11.0	11.7	32	10.0	12.6	32	− 8.8 (− 13.0 to − 4.6)	0.70	− 9.8 (− 14.1 to − 5.6)	0.78
Psychosocial impairment	WD2-12 subscale (0–100)	23.8	15.4	33	14.3	12.8	32	13.3	13.8	32	− 9.7 (− 14.6 to − 4.9)	0.67	− 10.8 (− 15.6 to − 5.9)	0.75
Mobility impairment	WD2-12 subscale (0–100)	14.5	7.3	33	12.6	6.2	32	12.6	7.3	32	− 6.8 (− 10.9 to − 2.7)	0.59	− 8.4 (− 12.6 to − 4.3)	0.65
Self-care impairment	WD2-12 subscale (0–100)	32.1	16.9	33	25.6	11.5	32	24.0	12.9	32	− 1.9 (− 4.1 to 0.2)	0.32	− 2.0 (− 4.2 to 0.2)	0.29
Screening score of at least 1														
Cardiopulmonary symptoms	PHQ-15 subscale (0–2)^a^	1.1	0.4	15	0.5	0.5	14	0.5	0.3	14	− 0.6 (− 0.8 to − 0.4)	1.16	− 0.5 (− 0.7 to − 0.3)	1.09
Fatigue symptoms	PHQ-15 subscale (0–2)^a^	1.3	0.4	25	0.8	0.6	25	0.9	0.5	25	− 0.5 (− 0.7 to − 0.3)	0.76	− 0.3 (− 0.6 to − 0.1)	0.61
Gastrointestinal symptoms	PHQ-15 subscale (0–2)^a^	1.2	0.4	18	0.9	0.4	18	0.9	0.6	18	− 0.4 (− 0.6 to − 0.1)	0.65	− 0.4 (− 0.6 to − 0.1)	0.68
Pain symptoms	PHQ-15 subscale (0–2)^ab^	1.2	0.4	17	0.7	0.7	17	0.8	0.6	17	− 0.4 (− 0.6 to − 0.2)	0.70	− 0.3 (− 0.6 to − 0.1)	0.63

Intention-to-treat estimates based on piecewise linear mixed effects models with a spline at the post-treatment assessment. *ASI-16* 16-item Anxiety sensitivity index, *3MFU* 3-month follow-up, *HAI-14* 14-item Health Anxiety Inventory, *PHQ-9* Patient Health Questionnaire 9, *PHQ-15* Patient Health Questionnaire 15 with subscales based on Witthöft et al. [49], *SSD-12* Somatic Symptom Disorder 12, *WD2-12* 12-item World Health Organization Disability Assessment Schedule 2 with subscales based on Axelsson et al. [56]

^a^Also measured on a weekly basis after weeks 1–7 in treatment, so that change over the main phase could be modelled using data from 9 assessments. Phrasings of these questionnaires were changed so as to concern the past week

^b^Curvilinear pattern of improvement as indicated by the fixed quadratic effect of main phase time improving model fit. For these outcomes, change scores for the Pre-Post main phase represent the sum of the time and time^2^ coefficients, and change for the Pre-3MFU period represent the sum of the time, time^2^ and follow-up-time coefficients

^c^Cohen’s *d* effect sizes calculated as the model-implied mean change divided by the observed standard deviation of change over the corresponding time period

### Feasibility aspect 7: adverse events and negative experiences

Five out of 32 participants (16%) reported at least one adverse event, most commonly increased anxiety or stress, which usually subsided by the end of treatment. On the NEQ-20, the proportion of endorsed items was 25% for the increase in symptoms, 11% for the perceived insufficient quality of treatment, 0% for dependency, 0% for stigma and 15% for hopelessness. There was no indication of serious adverse events. See Supplement 1 for details.

## Discussion

In preparation for a potential randomised controlled trial, we evaluated the feasibility of a unified Internet-delivered exposure therapy for individuals with undifferentiated DSM-5 SSD: a persistent and clinically significant reactivity or preoccupation with somatic symptoms regardless of somatic symptom domain and combination of unwanted emotions. We found the intervention to be feasible in the sense that individuals with a wide spectrum of physical symptoms showed interest, rated the treatment as credible and adhered to the treatment protocol. We saw large reductions in subjective somatic symptom burden and symptom preoccupation, without serious adverse events. Unified interventions that can suit many forms of distress related to physical symptoms may be easier to disseminate for example in the primary health care setting, and may focus on more than one symptom domain at a time. The Internet-delivered format also ensures access to treatment, as little time is needed from the therapist, treatment is flexible in time and place and there is a low threshold for health care seeking.

Strengths of this study include the systematic baseline assessment in collaboration with a general practitioner, the evaluation of several key aspects of feasibility and the precise estimation of within-group change with a high degree of data retention. Limitations include the lack of control group which implies that we cannot determine if change in the efficacy outcomes were caused by participant engaging in exposure. Within-group effects were probably on the conservative side, considering that some participants may have contracted covid-19, and the covid-19 restrictions during this study may have made it more difficult to conduct certain exposure exercises. Another limitation is that, for pragmatic reasons, the participants were self-selected, which could indicate a highly motivated sample. Moreover, the participants were highly educated with a low average level of functional impairment, which implies that we know little about feasibility in patient groups where educational attainment is low and functional impairment is high.

Despite all participants having a diagnosis of SSD, from the viewpoint of clinicians used to working with symptom preoccupation in specific somatic diseases or syndromes, the treatment could be described as transdiagnostic. Though this small feasibility trial does not allow us to make distinctions between participants based on their somatic diagnoses or syndromes, in larger samples, the transdiagnostic nature of this sort of intervention may serve as a strength precisely because it allows for the direct comparison of outcomes over different patient groups that report a high degree of distress related to their physical symptoms. Overall, results were similar to exposure-based therapies for more restricted populations with distress related to physical symptoms with regard to treatment credibility [26, 29, 34, 40], working alliance [34, 40], satisfaction [11, 21, 22, 26, 29, 34] and adverse events [21, 22, 24, 26, 29, 34, 40]. Tentatively, within-group effects on somatic symptoms and symptom preoccupation were slightly smaller, possibly owing to the transdiagnostic approach, the relatively short duration of 8 weeks, or the fact that this study of exposure-based treatment was conducted during the covid-19 pandemic. Effects were also relatively similar to other interventions which have a similar target group but do not focus as heavily on conventional exposure exercises, notable examples being acceptance and commitment therapy [64, 65], multicomponent strains of cognitive behaviour therapy [66, 67] and emotional awareness and expression therapy [68]. In the next few years, knowledge about treatment approaches suitable for a wide spectrum of patients who experience distress related to somatic symptoms is likely to improve, considering that several other such protocols appear to be in development [69–71]. We also note that in this field, there are interesting differences in the sampling method used in different trials. While some recruit a broad spectrum of individuals with functional somatic syndromes [72], other works centre around DSM-5 somatic symptom disorder [4], and still other around ICD-11 bodily distress syndrome [73] (formerly “disorder” [42]). An important topic of further enquiry will be to evaluate which of these approaches that is most helpful, for whom, and why.

There is a need to explore if this form of unified exposure-based treatment can achieve acceptability also in the relevant routine care contexts such as primary care, and if so, how much various patient groups have to gain from being enrolled in an exposure-based treatment developed for their specific somatic disease or syndrome (such as anxiety in asthma, atopic dermatitis, atrial fibrillation and so on) rather than a treatment intended to suit a broader spectrum of patients with distress related to somatic symptoms (as evaluated here). Though there are many conceivable models for the implementation of this type of intervention in routine clinical care, having the mental health clinician working closely in tandem with the general practitioner is probably pivotal for credibility and patient safety in the primary health care setting. There is also a need to investigate more closely whether the population that would benefit from this form of unified exposure-based treatment is best captured by DSM-5 SSD, and if so under what precise operationalisation [74], or some other criteria. Our clinical experience from having assessed the controversial SSD B criteria and attempting to determine whether behaviours are excessive and thus pathological [74–76] in this and previous studies [34, 46, 77] is that this tends to be relatively straightforward in certain populations such as for many patients in apparently good health suffering from high levels of health anxiety, but that the diagnostic procedure could be considerably less reliable (i) in conditions with a controversial aetiology, (ii) when symptoms are inferred solely from the behaviour of the patient (as opposed to objective measures) and (iii) in conditions where a certain level of behavioural adaptation to symptoms is widely culturally sanctioned (such as in many chronic pain and chronic fatigue conditions) [78]. There could be benefits of a longer treatment, as clinicians and many participants seemed to agree that the treatment was shorter than ideal. This exposure-based treatment was also unusual in that it incorporated no additional treatment components over and above the psychoeducation and self-monitoring necessary for exposure. It may be beneficial to add acceptance strategies [79], behavioural activation [80], mindfulness exercises [36], the naming of emotions [11] or emotional regulation techniques [26] to the treatment.

## Conclusion

Delivering a unified Internet-delivered exposure-based treatment for individuals with SSD regardless of somatic symptom domain or type of preoccupation with symptoms appears to be feasible.

## Supplementary Information

**Additional file 1.** Supplement 1: Further information about questionnaires, adverse events, max-min values and pre-post correlations, and the impact of the covid-19 pandemic.

## Data Availability

The data analysed during the current study are not publicly available due to Swedish and European Union (EU) data protection and privacy legislation but can be requested from the corresponding author on reasonable request.

## Abbreviations

ASI-16: 16-Item Anxiety Sensitivity Index
CSQ-8: 8-Item Client Satisfaction Questionnaire
DSM-5: Statistical Manual of Mental Disorders 5
HAI-14: 14-Item Health Anxiety Inventory
NEQ-20: 20-Item Negative Effects Questionnaire
PHQ-9: Patient Health Questionnaire 9
PHQ-15: Patient Health Questionnaire 15
SSD: Somatic Symptom Disorder (i.e. the DSM-5 diagnosis)
SSD-12: Somatic Symptom Disorder 12 (i.e. the questionnaire)
WAI-6: 6-Item Working Alliance Inventory
WD2-12: 12-Item World Health Organization Disability Assessment Schedule 2
