# Supplementary material for: A unified Internet-delivered exposure treatment for undifferentiated somatic symptom disorder: single-group prospective feasibility trial
Source: Pilot Feasibility Stud. 2022 Jul 19;8:149. doi: 10.1186/s40814-022-01105-0 (PMC9294766; doi:10.1186/s40814-022-01105-0)
Supplement: Supplementary file 1 — Additional file 1. Supplement 1: Further information about questionnaires, adverse events, max-min values and pre-post correlations, and the impact of the covid-19 pandemic. [file 40814_2022_1105_MOESM1_ESM.pdf]

Supplement 1: A unified exposure treatment for undifferentiated somatic symptom disorder

Questionnaire details and subscales

The internal consistency of the Patient health questionnaire 15 (without item 4) was good (α=0.82) for the conventional version and adequate (α=0.73) for the modified version. Subscales of the 20-item Negative effects questionnaire were based on Rozental et al. (2016) [1], subscales of the Patient health questionnaire 15 were based on Witthöft et al. (2013) [2] and subscales of the 12-item World health organisation disability assessment schedule 2 were based on Axelsson et al. (2017) [3]. See Table S1.

Table S1. Scoring of subscales

Questionnaire	Subscale			
	Name	Theoretical range	Items used for scoring	Scoring
NEQ-20	Increase in symptoms	0-100% / 0-4	1-4, 6-8, 10	Proportion of portal items endorsed / mean severity rating of endorsed items
NEQ-20	Insufficient quality of treatment	0-100% / 0-4	15-20	Proportion of portal items endorsed / mean severity rating of endorsed items
NEQ-20	Dependency on treatment	0-100% / 0-4	14	Proportion of portal items endorsed / mean severity rating of endorsed items
NEQ-20	Stigma	0-100% / 0-4	9, 11	Proportion of portal items endorsed / mean severity rating of endorsed items
NEQ-20	Increased hopelessness	0-100% / 0-4	5, 12-13	Proportion of portal items endorsed / mean severity rating of endorsed items
PHQ-15	Cardiopulmonary symptoms	0-2	6-7, 9-10[b]	(item_6 + item_7 + item_9 + item_10) / 4
PHQ-15	Fatigue symptoms	0-2	14-15[b]	(item_14 + item_15) / 2
PHQ-15	Gastrointestinal symptoms[a]	0-2	1, 12-13[b]	(item_1 + item_12 + item_13) / 3
PHQ-15	Pain symptoms	0-2	2-3, 5[b]	(item_2 + item_3 + item_5) / 3
WD2-12	Psychosocial impairment	0-100	1-12	(((-0.008×item_1 + 0.540×item_2 + 0.740×item_3 + 0.848×item_4 + 0.652×item_5 + 0.683×item_6 + -0.003×item_7 + -0.068×item_8 + 0.078×item_9 + 0.704×item_10 + 0.618×item_11 + 0.814×item_12) + 0.316) / (22.708 + 0.316))*100
WD2-12	Mobility impairment	0-100	1-12	(((0.877×item_1 + 0.264×item_2 + 0.035×item_3 + 0.042×item_4 + 0.074×item_5 + -0.040×item_6 +

				0.597×item_7 + 0.132×item_8 + -0.126×item_9 + -0.164×item_10 + -0.101×item_11 + 0.037×item_12) + 1.724) / (8.232 + 1.724))*100
WD2-12	Self-care impairment	0-100	1-12	(((-0.097×item_1 + -0.027×item_2 + -0.002×item_3 + -0.046×item_4 + -0.087×item_5 + -0.010×item_6 + 0.329×item_7 + 0.893×item_8 + 0.909×item_9 + 0.167×item_10 + 0.128×item_11 + -0.059×item_12) + 1.312) / (9.704 + 1.312))*100

NEQ-20, 20-item Negative effects questionnaire; PHQ-15, Patient health questionnaire 15; WD2-12, 12-item World health organisation disability assessment schedule 2.

[a] We made one change from Witthöft et al. (2013) in that we excluded the “pain or problems during sexual intercourse” item from the gastrointestinal subscale.

[b] The PHQ-15 item numbering differs somewhat in the literature. This is the same ordering of items that was used in the original publication (1 corresponding to a and so on) [4].

Feasibility aspect 6: Adverse events and negative experiences

Five out of 32 participants (16%) reported at least one adverse event and one participant (3%) reported two adverse events. Participants rated how much each event first had affected them, and also how much it still affected them at post-treatment, on a scale from 0 ("did/does not affect me at all") to 4 ("affected/s me very negatively"). Reported adverse events were: increased anxiety, stress or distress (n=3, initial distress: 1, 2, 4; post-treatment; 0, 2, 4), a negative outlook on the future caused by poor treatment outcome (n=1; initial: 4; post-treatment: 4), distress caused by conflicting information on how to optimise the effect of mediation (n=1; initial: 1; post-treatment: 1) and headache during interoceptive exposure (n=1; initial: 1; post-treatment: 0). There was no indication of serious adverse events.

On the NEQ-20, participants endorsed a mean of 3.1 (SD=3.7) out of 20 negative experiences that may occur during psychological treatment. Participants indicated how much each negative experience had affected them on a scale from 0 ("not at all") to 4 ("extremely"), and if they thought that the experience had been caused by the treatment. The proportion of endorsed items was 25% for the increase in symptoms (19 participants; mean severity: 1.7 [SD=0.8], due to treatment: 72%), 11% for the perceived insufficient quality of treatment (10 participants; mean severity: 1.2 [SD=0.8], due to treatment: 96%), 0% for dependency, 0% for stigma and 15% for hopelessness (6 participants; mean severity: 2.1 [SD=0.8], due to treatment: 100%).

Table S2. Observed max and min, and pre-post correlation, as based on observed values

Outcome	Measure (theoretical range)	Pre-treatment			Post-treatment			3 months			Pre-post corr.
		Max	Min	n	Max	Min	n	Max	Min	n	r
Subjective somatic symptom burden	PHQ-15 (0-30)	1	20	33	0	21	32	0	22	32	0.58
Cardiopulmonary symptoms	PHQ-15 subscale (0-2)	0	1.75	33	0	1.25	32	0	1.25	32	0.35
Fatigue symptoms	PHQ-15 subscale (0-2)	0	2	33	0	2	32	0	2	32	0.37
Gastrointestinal symptoms	PHQ-15 subscale (0-2)	0	2	33	0	2	32	0	2	32	0.50
Pain symptoms	PHQ-15 subscale (0-2)	0	2	33	0	2	32	0	2	32	0.56
Symptom preoccupation	SSD-12 (0-48)	7	47	33	3	45	32	0	46	32	0.52
Anxiety sensitivity	ASI-16 (0-64)	7	52	33	3	39	32	0	51	32	0.48
Health anxiety	HAI-14 (0-42)	12	39	33	7	40	32	3	39	32	0.60
General anxiety	GAD-7 (0-21)	1	21	33	1	19	32	0	18	32	0.63
Depression symptoms	PHQ-9 (0-27)	0	18	33	0	18	32	0	20	32	0.63
Overall functional impairment	WD2-12 (0-100)	0	60	33	0	48	32	0	58	32	0.56

ASI-16, the 16-item Anxiety sensitivity index; HAI-14, the 14-item Health anxiety inventory; PHQ-9, the Patient health questionnaire 9; PHQ-15, the Patient health questionnaire 15 with subscales based on Witthöft et al. (2013) [49]; SSD-12, the Somatic symptom disorder 12; WD2-12, the 12-item World health organisation disability assessment schedule 2 with subscales based on Axelsson et al. (2017) [56].

Possible impact of the covid-19 pandemic

The main phase of this feasibility study was conducted in the autumn of 2020, during the covid-19 pandemic. As is illustrated in Table S3, there was a significant increase in the perceived negative effects of the pandemic on mental health, from pre- to post-treatment (t=-3.1--2.4, df=31, P=0.004-0.021). We summed these three items and conducted a non-planned exploratory inferential analysis where we tested if the resulting pre-treatment variable, indicative of total mood disturbance due to the pandemic at baseline, moderated the improvement seen on the PHQ-15. The coefficient was 0.0 (95% CI: -0.4 to 0.4). Also, the correlation between pre-post change in pandemic-related mood disturbance and pre-post change in the PHQ-15 was small (r=0.05). In summary, though pandemic-related distress increased over the treatment period, there was apparently no effect of this on the improvement seen in subjective somatic symptom burden as measured using the PHQ-15. This said, some participants may have contracted covid-19 (data not collected), and it is also likely the case that the covid-19 restrictions made it more difficult to conduct certain exposure exercises, which means that the efficacy outcomes reported from this study are likely to be conservative. Such effects of the pandemic may conceivably contribute to explain the curvilinear pattern of change of a kind that is otherwise seldom seen in this field.

Table S3. Three items concerning the covid-19 pandemic, each scored 0-6

	During the past 2 weeks, how worried have you been with regard to covid-19 (the corona virus)?								
	Not at all worried		Worried to some degree		Much worried		Extremely worried	M (SD)	r with the PHQ-15
Pre-treatment	7 (21%)	5 (15%)	15 (45%)	1 (3%)	3 (9%)	2 (6%)	0 (0%)	1.8 (1.4)	0.12
Post-treatment	2 (6%)	4 (13%)	11 (34%)	8 (25%)	4 (13%)	3 (9%)	0 (0%)	2.5 (1.3)	-0.06
3 months	7 (22%)	6 (19%)	10 (31%)	4 (13%)	3 (9%)	1 (3%)	1 (3%)	1.9 (1.6)	-0.17
	Think back to your distress related to health and physical symptoms over the past 2 weeks. To what extent do you believe that this distress was due to the covid-19 pandemic and its consequences (e.g., the risk of contagion, social distancing, economic implications)?								
	I do *not at all* think that my distress related to health and symptoms is more substantial due to the ongoing pandemic. The level of distress had probably been the same (or		I think that my distress related to health and symptoms is *to some degree* more substantial due to the ongoing pandemic. The level of distress had to some		I think that my distress related to health and symptoms is *much* more substantial due to the ongoing pandemic. The level of distress had been much		I think that my distress related to health and symptoms is *extremely much* more substantial due to the ongoing pandemic. The level of distress had been extremely	M (SD)	r with the PHQ-15

	worse) in the past 2 weeks without the covid-19 pandemic.		degree been lower in the past 2 weeks without the covid-19 pandemic.		lower in the past 2 weeks without the covid-19 pandemic.		much lower in the past 2 weeks without the covid-19 pandemic.		
Pre-treatment	18 (55%)	6 (18%)	7 (21%)	0 (0%)	1 (3%)	1 (3%)	0 (0%)	0.9 (1.2)	-0.13
Post-treatment	9 (28%)	8 (25%)	8 (25%)	3 (9%)	3 (9%)	1 (3%)	0 (0%)	1.6 (1.4)	0.05
3 months	13 (41%)	3 (9%)	9 (28%)	5 (16%)	2 (6%)	0 (0%)	0 (0%)	1.4 (1.3)	0.01
	Over the past 2 weeks, to what extent has your general mood been poor due to the ongoing covid-19 pandemic (the corona virus) and its consequences (e.g., the risk of contagion, social distancing, economic implications)?								
	I do *not at all* think that I feel bad due to covid-19. I had felt the same (or worse) in the past 2 weeks without the covid-19 pandemic and its consequences.		I think that my mood is worse *to some degree* due to covid-19. I had to some degree felt better in the past 2 weeks without the covid-19 pandemic and its consequences.		I think that my mood is *much* worse due to covid-19. I had felt much better in the past 2 weeks without the covid-19 pandemic and its consequences.		I think that my mood is *extremely much* worse due to covid-19. I had felt extremely much better in the past 2 weeks without the covid-19 pandemic and its consequences.	M (SD)	r with the PHQ-15
Pre-treatment	15 (45%)	4 (12%)	12 (36%)	1 (3%)	1 (3%)	0 (0%)	0 (0%)	1.1 (1.1)	0.02
Post-treatment	8 (25%)	9 (28%)	8 (25%)	4 (13%)	3 (9%)	0 (0%)	0 (0%)	1.5 (1.3)	0.10
3 months	8 (25%)	7 (22%)	7 (22%)	4 (13%)	5 (16%)	1 (3%)	0 (0%)	1.8 (1.5)	0.11

PHQ-15, the Patient health questionnaire 15.

References

1. Rozental A, Kottorp A, Boettcher J, Andersson G, Carlbring P. Negative Effects of Psychological Treatments: An Exploratory Factor Analysis of the Negative Effects Questionnaire for Monitoring and Reporting Adverse and Unwanted Events. PLoS One. 2016;11(6):e0157503.
2. Witthöft M, Hiller W, Loch N, Jasper F. The latent structure of medically unexplained symptoms and its relation to functional somatic syndromes. Int J Behav Med. 2013;20(2):172-83.
3. Axelsson E, Lindsäter E, Ljótsson B, Andersson E, Hedman-Lagerlöf E. The 12-item Self-Report World Health Organization Disability Assessment Schedule (WHODAS) 2.0 Administered Via the Internet to Individuals With Anxiety and Stress Disorders: A Psychometric Investigation Based on Data From Two Clinical Trials. JMIR Ment Health. 2017;4(4):e58.
4. Kroenke K, Spitzer RL, Williams JB. The PHQ-15: validity of a new measure for evaluating the severity of somatic symptoms. Psychosom Med. 2002;64(2):258-66.
